# Temperature regulation mechanisms of diapause in *Coridius chinensis* revealed by multi-omics integration: coordinated responses of Brain-Gut-Fat Body

**DOI:** 10.3389/fmicb.2026.1810191

**Published:** 2026-07-09

**Authors:** Yantao Pang, Yajuan Chen, Qi Huang, Fangdong You, Richan Fang, Mingyang Geng, Xiaohuan Ke, Jianmin Tang, Jiating Ling, Youlin Cheng, Cuixia Zhao, Xianyu Deng, Jun Guo, Chunhui Miao

**Affiliations:** 1 Institute of Sericulture and Apiculture, Yunnan Academy of Agricultural Sciences, Mengzi, China; 2 Faculty of Life Science and Technology, Kunming University of Science and Technology, Kunming, Yunnan, China; 3 Kunming Women and Children’s Health Hospital, Kunming, Yunnan, China; 4 Yunnan Provincial Animal Husbandry Station, Kunming, Yunnan, China; 5 Yili Kazakh Autonomous Animal Husbandry General Station, Yining, China; 6 Yunnan Zhongfeng Technology Development Co., LTD., Kunming, Yunnan, China; 7 Department of Modern Agriculture, Zunyi Vocational and Technical College, Zunyi, China

**Keywords:** brain-gut axis, *Coridius* ***chinensis***, diapause, enteric microorganism, intestinal flora

## Abstract

Diapause in *Coridius chinensis* is a complex survival strategy that enables them to survive under prolonged cold stress. To elucidate the mechanisms of temperature regulation during diapause, we conducted multi-omics analyses, including gut metagenomics, brain transcriptomics, and fat body metabolomics, under both normal (25 °C) and diapause conditions (4 °C). Gut microbiome analysis revealed an extreme polarization during diapause, dominated by the endosymbionts *Pantoea endophytica* (52%) and *Rickettsia bellii* (47.4%), while functional microbiota such as *Pantoea* and *Dietzia* were significantly reduced. This shift suggests a trade-off where microbial metabolic diversity is sacrificed in favor of intracellular symbionts that may regulate host mitochondrial activity and suppress energy consumption. Brain transcriptomic analysis indicated a downregulation of neural signaling pathways related to feeding suppression, stress resistance, and circadian rhythm regulation. Fat body metabolomics identified the coordinated activation of 13 core pathways that link energy storage with stress adaptation, with dynamic changes ranging from rapid stress responses (0–300 AU) to energy storage dominance (300–500 AU), and finally to a state of homeostasis (>500 AU). Notably, dysregulated choline metabolism was significantly correlated with necrotic features (*r* = 0.78, *p* < 0.001), while catecholamine biosynthesis derived from tyrosine emerged as a corrective pathway, revealing the mechanistic link between metabolic flexibility and survival. Adults primarily utilize plants within the Cucurbitaceae, Fabaceae, and Solanaceae families as hosts, underpinned by long-standing folk traditions in specific localities regarding their dietary consumption or therapeutic application.

## 1 Introduction

The medicinal stink bug, *Coridius chinensis* (class Insecta, order Hemiptera, family Dinidoridae), represents a traditional resource insect utilized for both nutritional and pharmacological purposes; contemporary medical research has substantiated its potent antibacterial and antitumour properties, alongside its significant health-promoting value (Wu et al., 2019). Its geographical distribution spans from East to Southwest China, extending to Taiwan, the Ryukyu Islands, and certain regions within Southeast Asia (Dallas and Gray, 1851). Adults primarily utilize plants within the Cucurbitaceae, Fabaceae, and Solanaceae families as hosts, underpinned by long-standing folk traditions in specific localities regarding their dietary consumption or therapeutic application. The overwintering sedentary behavior of adults, coupled with their selection of microhabitats for hibernation (such as rock crevices and leaf litter), exerts a profound influence on both local ecosystems and the management of agricultural pests (Zhang et al., 2023).

Diapause constitutes a state of physiological developmental arrest or metabolic suppression evolved by insects and other arthropods to survive seasonally adverse environmental conditions (Denlinger, 2023). Distinct from transient quiescence, diapause is a programmed, stage-specific physiological state, typically determined during precise developmental windows by the interplay between environmental cues–including photoperiod, temperature, and nutritional signals–and endogenous rhythms, and is subsequently mediated via complex hormonal networks (Easwaran and Montell, 2023). Diapause enhances individual survivorship during periods of frigidity, desiccation, or nutritional scarcity; simultaneously, it facilitates temporal synchronization through deferred reproduction, thereby maintaining population propagation and distributional stability amidst interannual environmental fluctuations (Numata and Shintani, 2023). Furthermore, diapause holds substantial significance within agro-ecology: numerous pests overwinter in a diapausing state, which dictates their phenological dynamics and the subsequent windows for control interventions in the ensuing year (Ni et al., 2025). Conversely, for resource insects or populations amenable to mass-rearing, elucidating and terminating diapause is pivotal for industrial-scale propagation and exploitation (Mashal et al., 2024).

Temperature serves as both a direct and indirect determinant of diapause; for numerous species, the confluence of abbreviated photoperiods and declining thermal gradients is perceived and integrated as “seasonal cues” to initiate the diapause programme. Conversely, in certain other taxa–particularly those originating from high-latitude or high-altitude populations–the phenomenon of “cold-inducible diapause” may manifest (Musolin and Numata, 2003). Substantial interspecific variation exists regarding sensory thresholds and sensitivity to thermal fluctuations; furthermore, temperature not only dictates induction but also governs metabolic rates and overwintering survivorship throughout the maintenance of diapause (Horikawa et al., 2024). Ambient temperature fluctuations orchestrate a hierarchical coupling network via the “Brain-Gut-Fat Body” axis, which collectively determines the induction, maintenance, and termination phases of diapause in *Coridius chinensis*. The underlying logic is that thermal inputs are perceived and integrated by thermosensory and circadian networks within the brain (TRP/TRPA1 and circadian clock genes), which subsequently modulate endocrine pathways involving neuropeptides, juvenile hormone (JH), and 20-hydroxyecdysone (20E) (Helfrich-Förster, 2024; Hutfilz, 2022). The gut microbiota exhibits sensitivity to both temperature and host endocrine signals; shifts in its functional pathways–such as the metabolism of short-chain fatty acids (SCFAs), tryptophan-indoles, and polyols–generate metabolic factors that enter the systemic circulation, providing feedback that influences host energy metabolism and immune homeostasis (Liu et al., 2016; Yasika and Shivakumar, 2025). The fat body serves as the primary hub for energy and cryoprotective metabolism (trehalose/glycerol and lipid mobilization); under the influence of JH and insulin-TOR-FOXO signaling, it remodels its metabolic and storage architecture to sustain the characteristic hypometabolism, stress resistance, and reproductive suppression essential for diapause (Hahn and Denlinger, 2011; Izadi, 2025). Synergy is established among these three components through feed-forward and feedback loops: thermo-neuroendocrine signals alter the gut ecological niche and metabolic flux, while gut-derived metabolites, in turn, modulate the signal sensitivity and metabolic set-points of both the brain and the fat body, ultimately shaping the diapause phenotype (Xu et al., 2012).

This study focuses on *Coridius chinensis* as the model organism, aiming to systematically elucidate the molecular and metabolic mechanisms underpinning low-temperature-induced diapause, predicated on the “Brain-Gut-Fat Body” multi-organ synergistic regulation hypothesis. By integrating multi-omics datasets with experimental validation, this research seeks to reveal the processes through which thermal information is perceived and integrated at a systemic level, and subsequently transduced into regulatory signals that dictate diapause phenotypes. Through the characterization of dynamic transcriptomic shifts within brain tissues during cold induction, we aim to screen thermosensory genes (e.g., TRP/TRPA1), circadian-related genes, and neuropeptide regulatory elements; this will facilitate the analysis of signal transmission to endocrine pathways–specifically juvenile hormone (JH) and 20-hydroxyecdysone (20E)–culminating in the construction of a robust “temperature-neuropeptide-hormone” regulatory model. Utilizing gut microbiome and metabolome profiling, we intend to uncover alterations in intestinal microbial architecture and metabolic functionality under cold stress; the objective is to decipher the roles of specific metabolites–including short-chain fatty acids (SCFAs), indole derivatives, and polyols–in host metabolic modulation and endocrine feedback, thereby defining the precise regulatory contributions of gut-derived factors during the induction and maintenance of diapause. Building upon an integrative analysis encompassing the brain transcriptome, gut micro-metabolome, and fat body omics, this study will establish a synergistic multi-organ network model for cold-induced diapause; furthermore, the functional verification of pivotal regulatory nodes–such as neuropeptides, metabolic enzymes, and microbial-derived metabolites–will be conducted to provide a comprehensive theoretical framework for understanding the systemic physiological mechanisms of insect diapause.

## 2 Materials and methods

### 2.1 Sample collection

Specimens of *Coridius chinensis* were excavated from rock piles in Zhaotong, with female adults exhibiting uniform body size selected as the experimental subjects. The insects were categorized into an ambient temperature group (CK group, non-diapausing) and a low-temperature treatment group (D group, diapause-induced). The CK group was reared at a constant 25 °C under continuous dark conditions for 7 days; conversely, the diapause group was subjected to a fluctuating thermal regime consisting of 12 h at 4 °C followed by 12 h at 25 °C daily, maintained for a total of 7 days. Following the 7-days treatment, the fat body, gut, and brain tissues were dissected and separately harvested, then immediately cryopreserved in an ultra-low temperature freezer at −80 °C.

### 2.2 Brain transcriptomics

Brain tissues were isolated from individuals of each treatment group; total RNA was subsequently extracted using the TRIzol reagent (Invitrogen, USA). RNA purity and concentration were quantified via NanoDrop 2000, while integrity was benchmarked using an Agilent 2100 Bioanalyzer (ensuring an RNA Integrity Number, RIN ≥ 7.0). Transcriptomic libraries were constructed using the Illumina TruSeq RNA Sample Preparation Kit and sequenced on the Illumina NovaSeq 6000 platform with a 150 bp paired-end (PE150) read length protocol. Raw sequencing data underwent quality assessment using FastQC (Chen et al., 2018), followed by the removal of adapter sequences and low-quality reads via Trimmomatic software (Bolger et al., 2014). The resulting clean reads were aligned to the reference genome using HISAT2 (or alternatively, *de novo* assembled via Trinity) (Kim et al., 2019). Gene expression levels were quantified and expressed as Fragments per Kilobase of transcript per Million mapped reads (FPKM). Differential expression analysis was performed using the DESeq2 package, with the significance thresholds established at a | log2 Fold Change| ≥ 1 and a False Discovery Rate (FDR) of <0.05 (Love et al., 2014). Gene Ontology (GO) annotation and enrichment analysis were conducted using the clusterProfiler R package, alongside Kyoto Encyclopaedia of Genes and Genomes (KEGG) pathway enrichment analysis, to identify signaling pathways associated with neuroregulation, hormone biosynthesis, and energy metabolism (Wang et al., 2010). Furthermore, Weighted Gene Co-expression Network Analysis (WGCNA) was employed to construct gene co-expression networks, facilitating the identification of gene modules and pivotal regulatory factors significantly correlated with specific diapause stages (Han et al., 2024).

### 2.3 Gut metagenomics

Midgut contents were harvested from individuals within each experimental group, and total genomic DNA was extracted utilizing the QIAamp DNA Stool Mini Kit (Qiagen, Germany). Following quantification and purity assessment via Qubit 2.0, metagenomic libraries–with an approximate insert size of 350 bp–were constructed and sequenced on the Illumina NovaSeq 6000 platform using a 150 bp paired-end (PE150) strategy (Versmessen et al., 2024). Raw sequencing data were subjected to quality evaluation using FastQC (Chen et al., 2018); subsequently, KneadData was employed to purge host-derived contaminant sequences, and MEGAHIT was utilized for the *de novo* assembly of clean reads (Li et al., 2015; Wirbel et al., 2019). Open Reading Frame (ORF) prediction was executed via Prodigal, whereas the construction of a non-redundant gene catalog was achieved using CD-HIT with a 95% identity threshold. Taxonomic assignment was performed against the NCBI non-redundant (NR) protein database, employing DIAMOND for high-throughput sequence alignment. Functional gene annotation was carried out using the Kyoto Encyclopedia of Genes and Genomes (KEGG) database (Kanehisa, 2000); furthermore, metabolic pathway profiles were reconstructed by integrating HUMAnN3 and MetaCyc analyses (Beghini et al., 2021). Significantly differentially abundant microbial biomarkers were identified through Linear Discriminant Analysis Effect Size (LEfSe) analysis, with their putative biological functionalities inferred in conjunction with metabolomic datasets (Segata et al., 2011).

### 2.4 Fat body metabolomics data acquisition

Solid tissue samples (100 mg) were transferred into 2 mL centrifuge tubes, each containing a single grinding bead with a 6 mm diameter. Metabolite extraction was performed using 800 μL of extraction solvent (methanol:water = 4:1, v/v) spiked with four internal standards, including L-2-chlorophenylalanine (0.02 mg/mL) (Jiang et al., 2025). The sample solutions were homogenized in a cryogenic tissue grinder for 6 min (at −10 °C and 50 Hz), followed by low-temperature ultrasonic extraction for 30 min (at 5 °C and 40 kHz). After being allowed to stand at −20 °C for 30 min, the mixture was centrifuged for 15 min (at 4 °C and 13000 *g*); the resulting supernatant was then aspirated and transferred into injection vials equipped with internal inserts for subsequent analysis (Qi et al., 2025). LC-MS analysis was executed utilizing an ultra-high-performance liquid chromatography system coupled with tandem Fourier transform mass spectrometry (UHPLC-Orbitrap Exploris 480, Thermo Fisher Scientific), facilitated by Majorbiomed Technology Co., Ltd., (Shanghai, China). Chromatographic separation was achieved by injecting a 3 μL sample onto an HSS T3 column (100 mm × 2.1 mm i.d., 1.8 μm) prior to mass spectrometric detection. Mobile phase A consisted of water/acetonitrile (95/5, v/v) containing 0.1% formic acid, while mobile phase B comprised acetonitrile/isopropanol/water (47.5/47.5/5, v/v/v) with 0.1% formic acid. The flow rate was maintained at 0.40 mL/min with a column temperature of 40 °C. Mass spectrometry (MS) parameters were configured for signal acquisition in both positive and negative ion scanning modes, covering a mass-to-charge ratio (m/z) range of 70–1050. The ion spray voltage was set at 3500 V for positive mode and −3000 V for negative mode; additional settings included a sheath gas flow of 50 arb, auxiliary heating gas at 13 arb, an ion source temperature of 450 °C, and cyclic collision energies of 20-40-60 V (Zhu et al., 2022).

Following data acquisition, raw LC-MS datasets were imported into Progenesis QI metabolomics processing software (Waters Corporation, Milford, USA) for baseline filtering, peak recognition, integration, retention time correction, and peak alignment, ultimately yielding a data matrix encompassing retention times, m/z values, and peak intensities. Simultaneously, MS and MS/MS spectral information were benchmarked against public databases, including HMDB and Metlin, as well as the Majorbiomed proprietary library, to achieve metabolite identification. The resulting data matrix was subsequently uploaded to the Majorbio Cloud Platform^1^ for downstream bioinformatic analysis (Han et al., 2024). Initial preprocessing of the data matrix was conducted as follows: the “80% rule” was applied to eliminate missing values, retaining only variables with non-zero values in at least 80% of samples within any single group, followed by the imputation of remaining gaps using the minimum value detected in the original matrix. To mitigate variances arising from sample preparation and instrumental instability, total sum normalization was applied to the response intensities of spectral peaks, resulting in a normalized data matrix. Variables with a relative standard deviation (RSD) exceeding 30% in quality control (QC) samples were excluded, and the data underwent log_{10} transformation to derive the final matrix for subsequent statistical analyses (He et al., 2024). Subsequently, the “ropls” package (Version 1.6.2) in R was employed to perform Principal Component Analysis (PCA) and Orthogonal Partial Least Squares-Discriminant Analysis (OPLS-DA) on the preprocessed matrix, with model stability evaluated via seven-fold cross-validation. The selection of significantly differential metabolites was determined based on the Variable Importance in Projection (VIP) values derived from the OPLS-DA model and Student’s *t*-test *p*-values; metabolites exhibiting a VIP > 1 and *p* < 0.05 were categorized as significantly differential (Lee and Lee, 2021).

## 3 Results

### 3.1 Brain transcriptomics reveal neuroendocrine regulatory mechanisms

**FIGURE 1:**
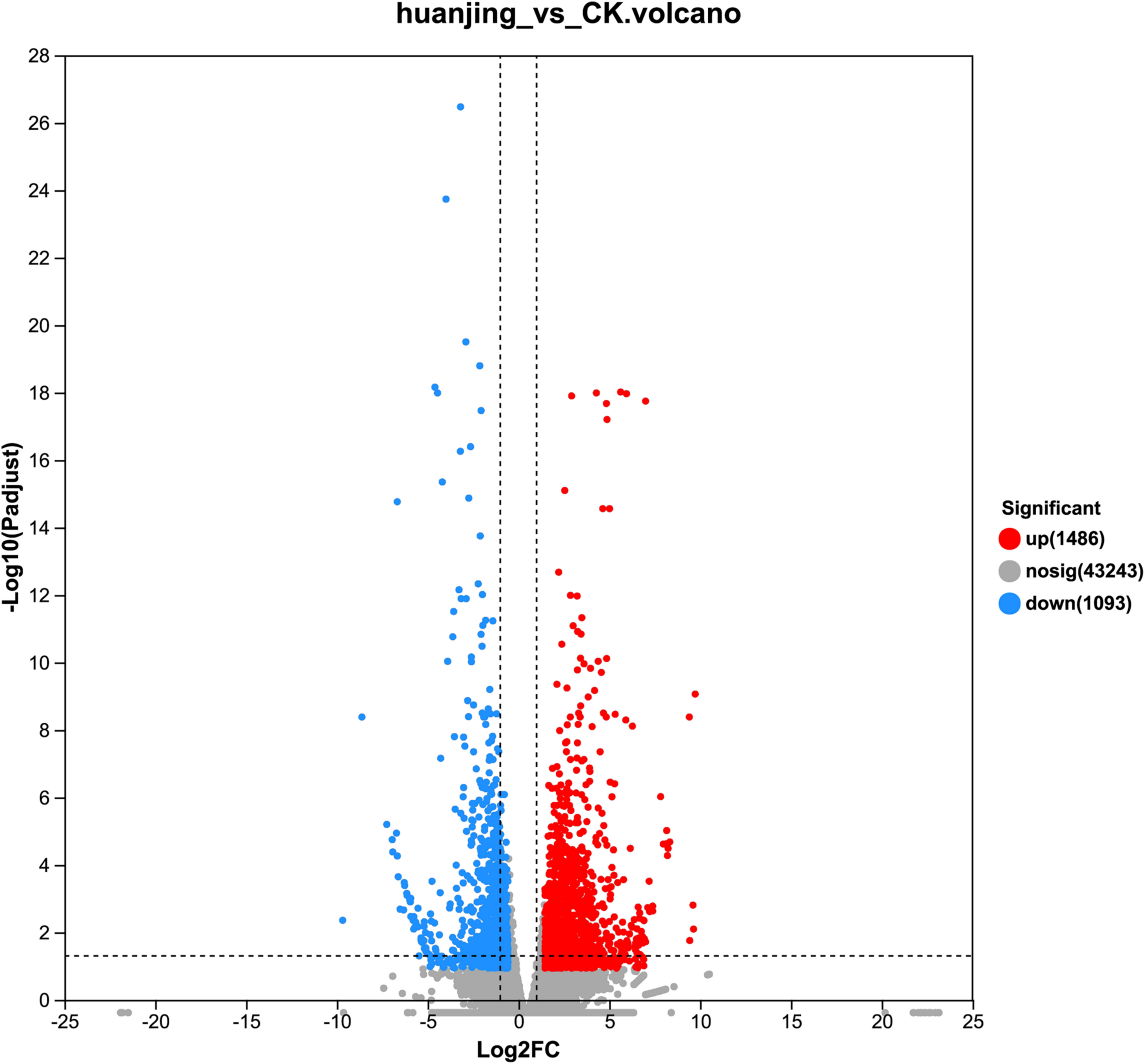
Volcano plot of differentially expressed genes (DEGs).

**FIGURE 2:**
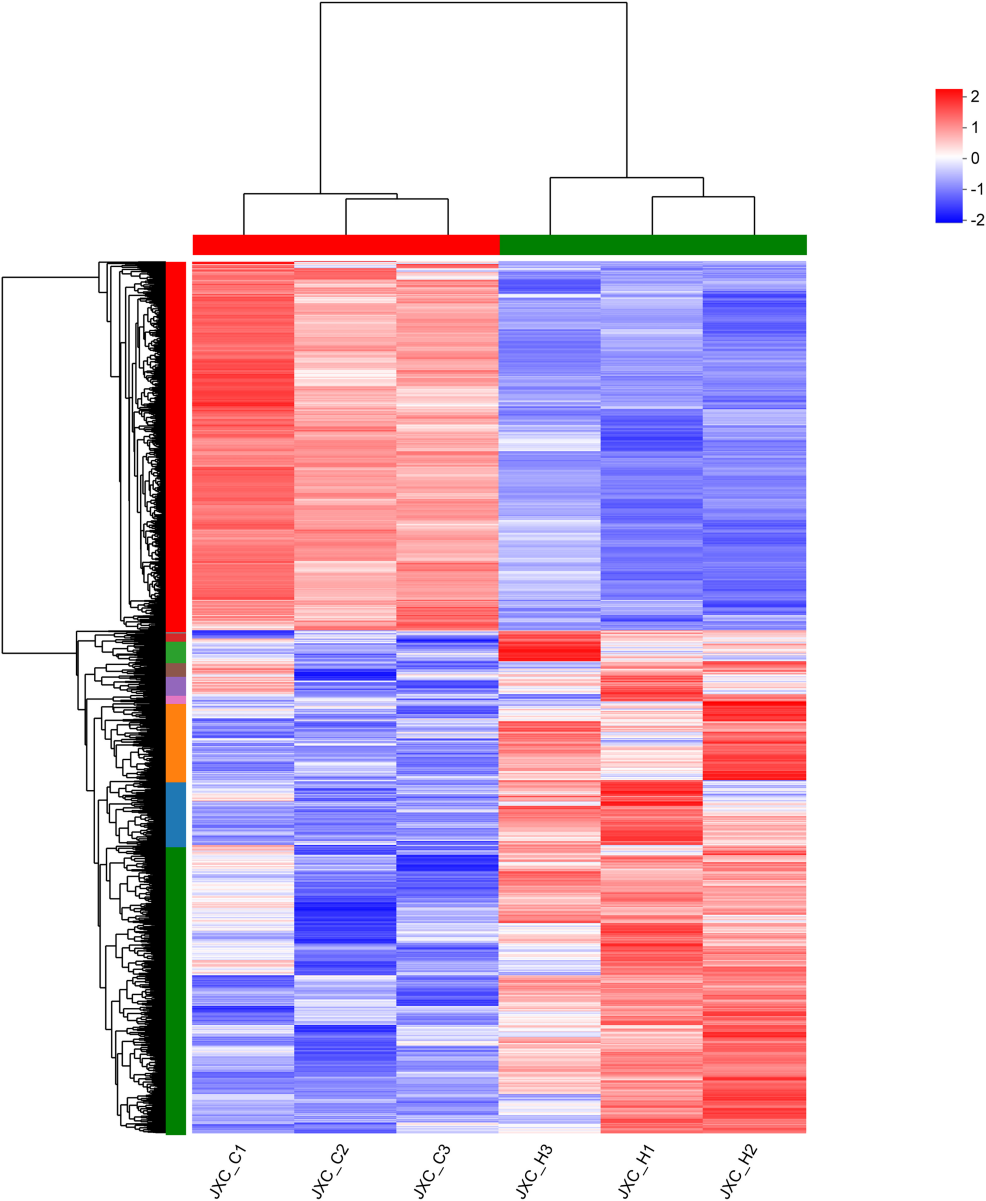
Hierarchical clustering heatmap of DEGs.

**FIGURE 3:**
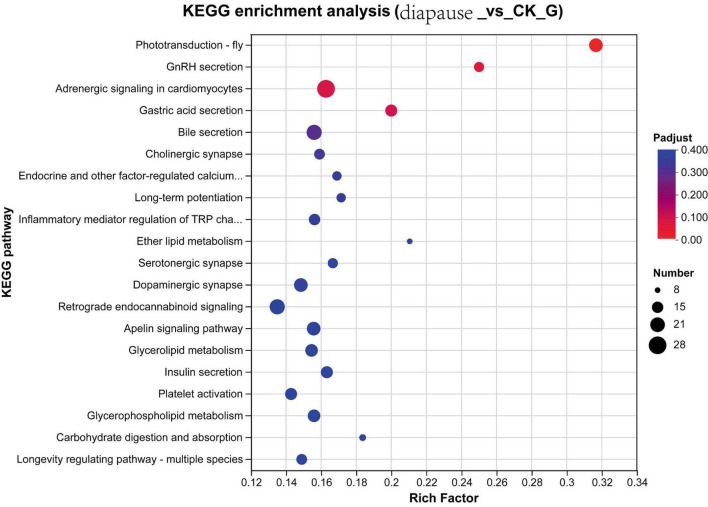
Kyoto Encyclopaedia of Genes and Genomes (KEGG) pathway enrichment bubble chart.

To elucidate the impact of low-temperature-induced diapause on the brain transcriptional regulatory networks of *Aspongopus chinensis*, RNA-seq and differential expression analyses were performed on brain tissues from both the diapause-induced (D) and ambient control (CK) groups. Following quantification via RSEM and normalization using DESeq2, a total of 45,822 expressed genes were identified, of which 2,579 exhibited significant differential expression between the two groups (| log2FC| ≥ 1, FDR < 0.05). Specifically, 1,486 genes were up-regulated and 1,093 were down-regulated, with differentially expressed genes (DEGs) accounting for approximately 5.6% of the total transcriptome (Figures 1–3). The volcano plot revealed that the number of significantly up-regulated genes slightly exceeded those down-regulated following cold exposure; this indicates that low-temperature stress triggers a broad transcriptional activation response, involving functional genes associated with neural signal transduction, energy metabolism, and thermosensation. Hierarchical clustering analysis (Figure 2) demonstrated a distinct separation in expression profiles between the diapause and control groups, with high intra-group consistency, suggesting that thermal treatment is the primary driver of transcriptional divergence. The heatmap categorized DEGs into two complementary clusters: the first cluster, significantly up-regulated in the diapause group, primarily comprised heat shock proteins (HSPs), transient receptor potential (TRP) channels, genes within the insulin/FOXO pathway, neurotransmitter receptors, and ligand-binding proteins (Rinehart et al., 2007; Ma et al., 2024). In contrast, the second cluster was down-regulated in the diapause group and largely associated with ribosomal assembly, glycolysis, and nucleotide metabolism, suggesting that cold-induced diapause is characterized by the suppression of anabolic metabolism alongside the activation of stress responses. Kyoto Encyclopaedia of Genes and Genomes (KEGG) pathway enrichment analysis identified 19 significantly enriched pathways (Padjust < 0.05). The DEGs were predominantly concentrated within three functional categories: neuroendocrine signaling pathways, including “Phototransduction–fly,” “GnRH secretion,” “Adrenergic signaling in cardiomyocytes,” and “Serotonergic” and “Cholinergic synapses.” The activation of these pathways implies that low temperatures may regulate the initiation and maintenance of diapause through a neurosensory-endocrine axis (TRP–JH–neurotransmitters). Energy and lipid metabolism pathways involved “Glycerophospholipid metabolism,” “Glycerolipid metabolism,” “Ether lipid metabolism,” and “Insulin secretion.” The observed up-regulation of lipid-related genes alongside the down-regulation of carbohydrate metabolism genes reflects a metabolic shift from carbohydrate utilization toward lipid reserve accumulation during diapause. Environmental perception and homeostasis maintenance pathways included “Inflammatory mediator regulation of TRP channels,” “Apelin signaling,” “Calcium signaling,” and “Long-term potentiation,” all of which are linked to thermosensation, cellular signal transduction, and adaptive stress responses. Statistically, the Rich Factor for the significantly enriched pathways ranged from 0.12 to 0.32, with “Phototransduction–fly,” “GnRH secretion,” and “Adrenergic signaling” exhibiting the most substantial enrichment (Padjust < 0.05). Combined with Gene Ontology (GO) functional annotations, the DEGs were predominantly involved in biological processes such as metabolic processes (GO:0008152), signal transduction (GO:0007165), responses to stimuli (GO:0050896), and developmental regulation (GO:0032502). In summary, low-temperature treatment profoundly altered the brain gene expression profile of *A. chinensis*, characterized by the activation of neurosensory and hormonal signaling pathways and the down-regulation of genes associated with biosynthesis and energy metabolism. These alterations collectively underscore a pivotal molecular mechanism of diapause: thermal signals are integrated via the brain’s neuro-endocrine network, inducing metabolic reprogramming and reproductive suppression, thereby providing the molecular foundation for diapause maintenance (Koštál, 2006).

**FIGURE 4:**
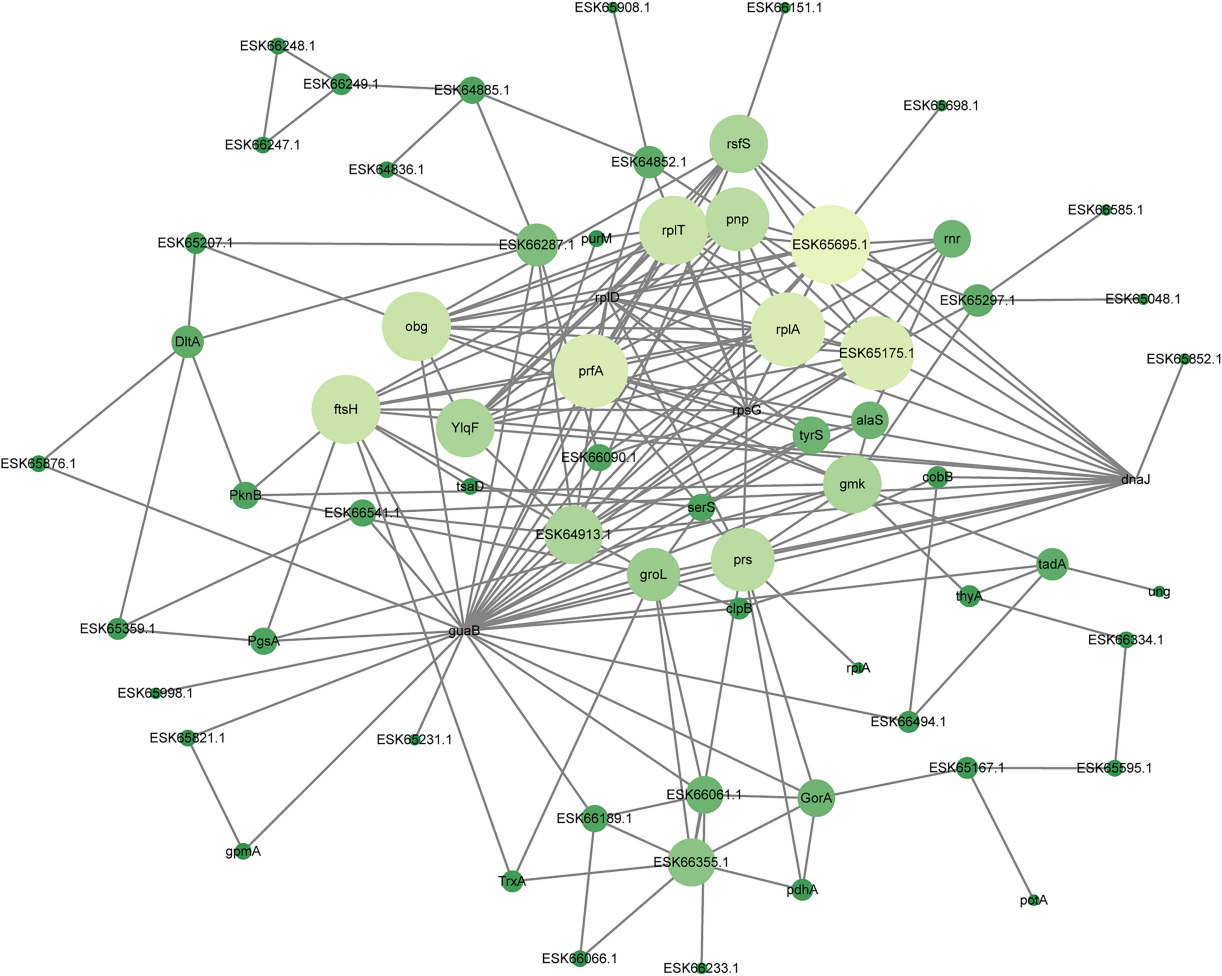
Protein-protein interaction (PPI) network.

Analysis of the protein-protein interaction (PPI) network identifies (Figure 4) rplD, rplT, rpsG, rplA, and rsfS as core proteins exhibiting the highest abundance; these proteins constitute a dense interaction network (with combined scores for rplD with rplT and rpsG reaching 0.999), collectively orchestrating ribosomal assembly and protein translation. Acting as a translational inhibitor, RsfS likely facilitates cellular adaptation to environmental stressors by inducing translational arrest under adverse conditions (Häuser et al., 2012). Furthermore, DnaJ operates synergistically with the molecular chaperone groL (combined score = 0.87) to preclude protein denaturation, thereby maintaining proteostasis during thermal shock and related stressors (Susin et al., 2006). DnaJ also interacts with ClpB, an ATP-dependent protease (combined score = 0.808), contributing to the proteolytic degradation of misfolded proteins (Doyle et al., 2007). Concurrently, the enzyme prs, which catalyses the formation of phosphoribosyl pyrophosphate (PRPP)–the rate-limiting step in purine biosynthesis–interacts with guaB (inosine-5′-monophosphate dehydrogenase) and purM (phosphoribosylformylglycinamidine cyclo-ligase) (combined scores > 0.78) to co-regulate guanine nucleotide synthesis (Ayoub et al., 2024; Hove-Jensen et al., 2017).

### 3.2 Gut metagenomics reveal microbial functional remodeling

The gut microbiota of *Coridius chinensis* remains relatively depauperate during diapause; however, as ambient temperatures rise (Figure 6), the microbial proportions undergo significant shifts characterized by the explosive proliferation of obligate intracellular symbionts and the concomitant contraction of metabolically functional microbial communities (Ferguson et al., 2018). The diapause group exhibited an extreme unipolarisation trend, where the phytosymbiont *Pantoea endophytica* (52%) and the rickettsial endosymbiont *Rickettsia bellii* (47.4%) attained absolute dominance; notably, the abundance of functional taxa such as *Pantoea* and *Dietzia* plummeted following low-temperature exposure. In contrast, the CK group maintained a more equitable microbial composition, wherein *Pantoea endophytica* (42.4%) and *Rickettsia bellii* (42.1%) established a dynamic equilibrium, complemented by the low-abundance coexistence of the stress-resistant *Dietzia* (4.6%) and the starch-degrading *Paenibacillus* (0.6%). These shifts suggest that the host may sacrifice metabolic diversity during diapause in exchange for energy homeostasis dominated by intracellular symbionts; specifically, the explosive expansion of *Rickettsia bellii* may reduce metabolic rates by modulating host mitochondrial functions, such as the inhibition of ATP synthesis. Furthermore, the collapse of plant polysaccharide-degrading bacteria (*Pantoea*) corroborates the physiological adaptation to the cessation of feeding behavior during the diapausing state (Liu et al., 2016).

**FIGURE 5:**
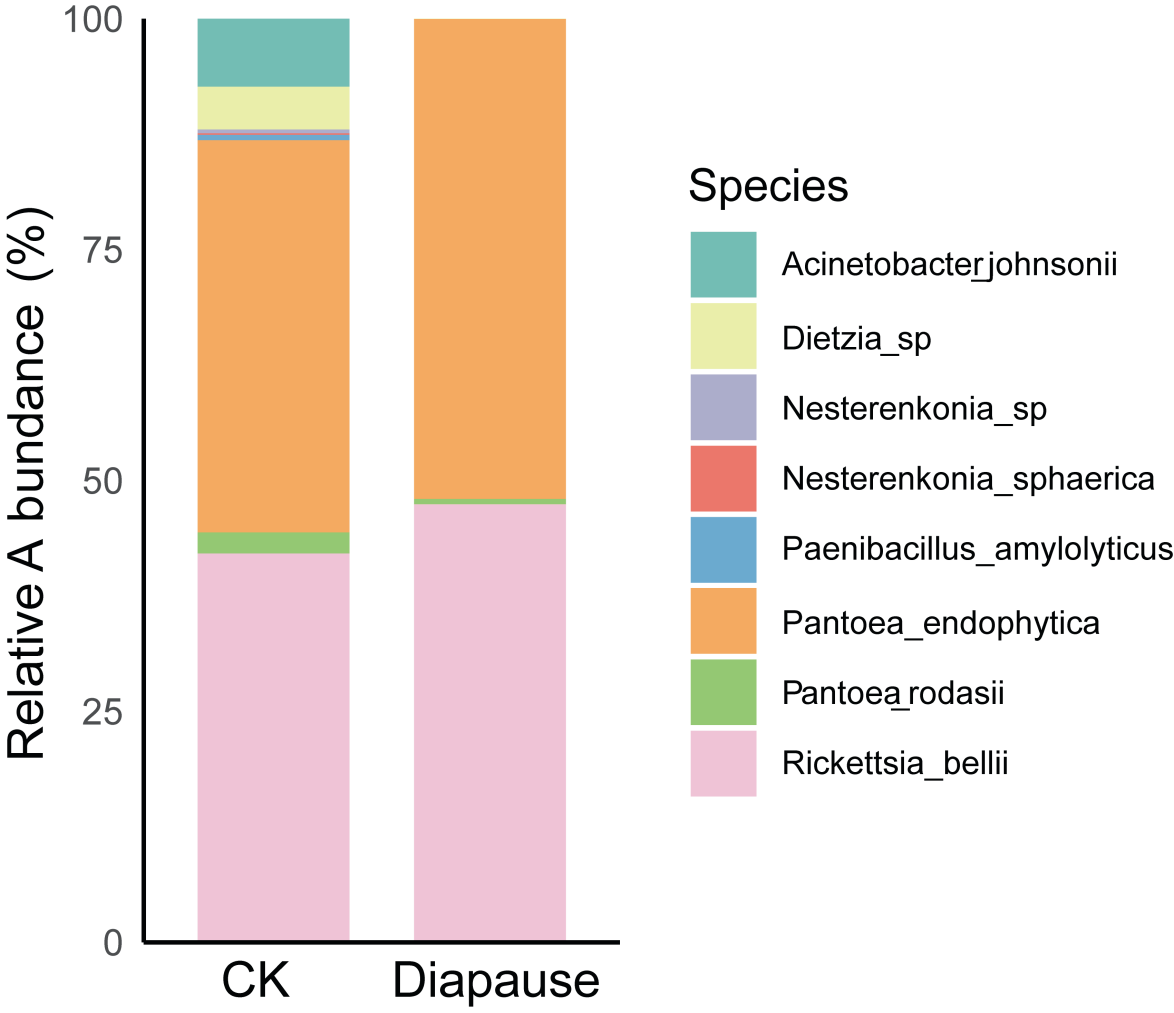
Relative abundance of microbial taxa.

Metagenomic results based on HUMAnN analysis reveal significant divergence in the functional composition of the *Coridius chinensis* gut microbiota across different thermal treatments (Figures 5, 7; Franzosa et al., 2018). A distinct separation between the two experimental groups was observed at both the gene family and pathway abundance levels, underscoring a systemic reconfiguration of the intestinal functional potential elicited by thermal exposure (Teslaa et al., 2023; Wang et al., 2024). Notably, the D group exhibited minimal intragroup variance alongside substantial intergroup differentiation, suggesting that temperature regulation exerts a consistent and stable shaping effect on the microbial functional networks. Within the D group samples, there was a pronounced enrichment in carbon metabolism pathways, including glycogen degradation (PWY-5941), glycolysis (PWY-1042), the non-oxidative phase of the pentose phosphate pathway (NONOXIPENT-PWY), and glucose-1-phosphate metabolism (GLUCOSE1PMETAB-PWY). The up-regulation of these metabolic routes indicates that the gut microbiota enhances energy mobilization and the generation of reducing power under diapausing temperatures, thereby providing essential energy substrates and NADPH sources for the host’s metabolic maintenance and stress-response mechanisms in frigid environments (Teslaa et al., 2023). Furthermore, pathways associated with purine and pyrimidine metabolism (PWY-6125, PWY-7221, PWY-7222, PWY-7228, PWY-7185, and PWY-5695) exhibited an upward trend in the D group; this suggests that during diapause, the microbial community facilitates nucleotide biosynthesis and turnover to support DNA/RNA repair and the metabolism of signaling molecules, thereby assisting in host cellular renewal and restorative processes (Rashida and Laxman, 2021; Teslaa et al., 2023).

**FIGURE 6:**
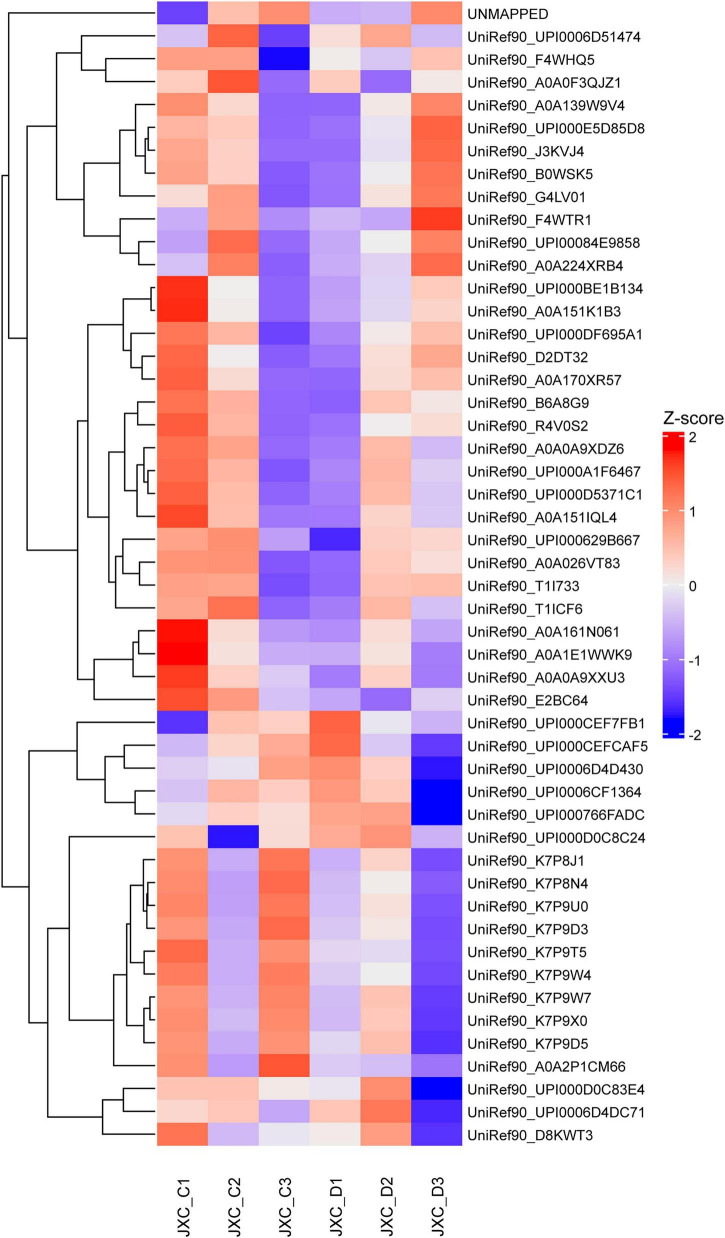
Heatmap of differential gene families.

**FIGURE 7:**
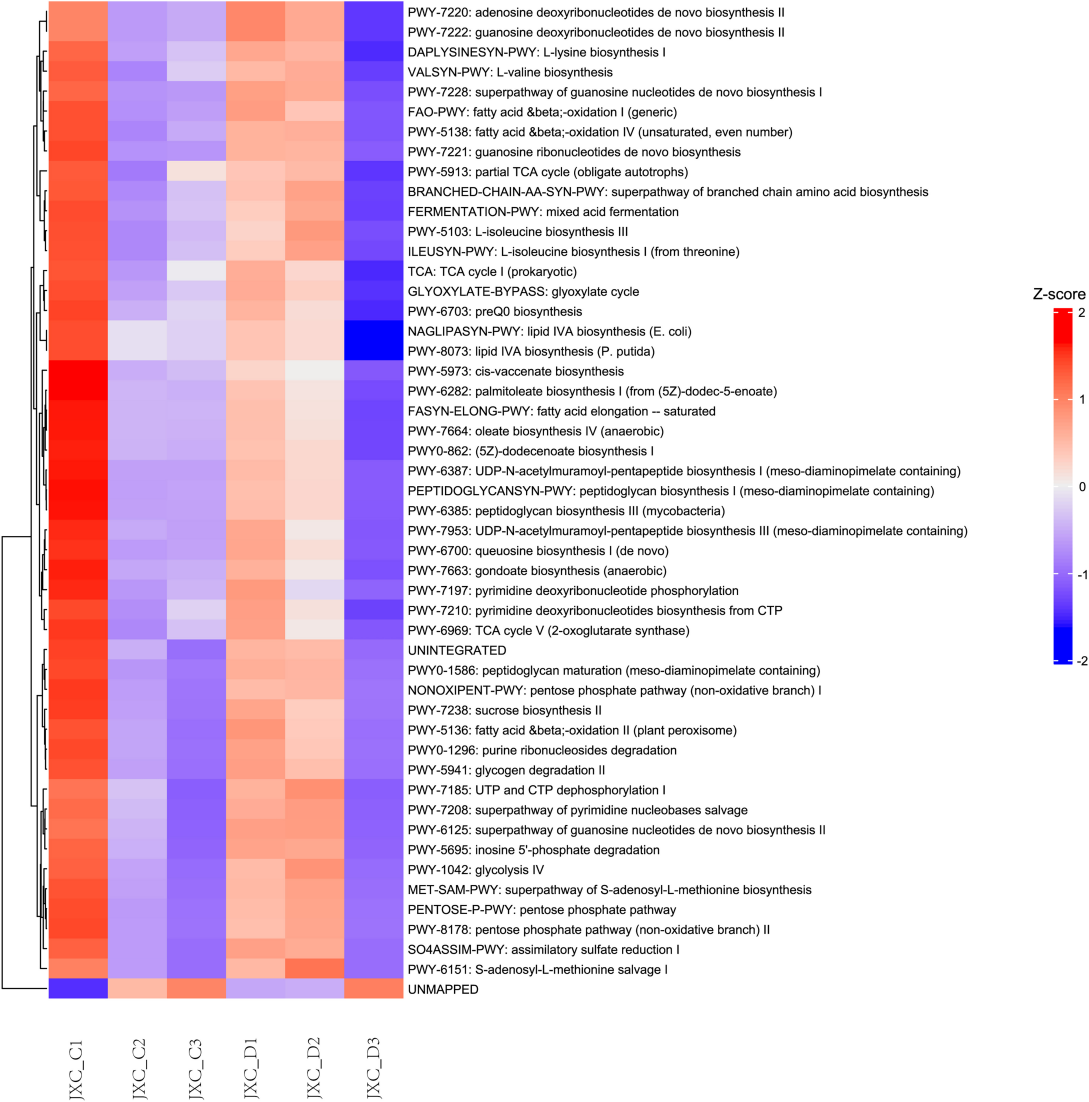
Heatmap of metabolic pathway abundance.

Under cold conditions, the D group exhibited a significant augmentation in the activity of pathways such as sulfate assimilation (SO4ASSIM-PWY) and S-adenosyl-L-methionine (SAM) recycling (PWY-6151), indicating that the gut microbiota facilitates sulfur metabolism and the methyl cycle during low-temperature diapause (Uchiyama et al., 2022). The intensification of these metabolic trajectories is likely linked to the host’s antioxidant defense mechanisms (e.g., glutathione biosynthesis) and epigenetic methylation regulation, which assist in mitigating cold-induced oxidative stress and maintaining the stability of gene expression (Raza et al., 2020). In contrast to the CK group, the D group showed a pronounced attenuation in the coverage of various fatty acid biosynthesis pathways (e.g., PWY-5973, PWY-7664, PWY-6282, and FASYN-ELONG-PWY), whereas pathways pertaining to peptidoglycan maturation and synthesis (PWY0-1586 and PEPTIDOGLYCANSYN-PWY) underwent up-regulation. This suggests that at diapausing temperatures, the microbial community suppresses the biosynthesis of unsaturated fatty acids and enhances cell wall stability, thereby contributing to the maintenance of membrane fluidity and structural integrity; such adaptations bolster the survival and ecological fitness of both the microbiota and the host in frigid environments (Tribelli and López, 2018).

Synthesizing the shifts in metabolic functionality, the following regulatory model is postulated: under diapausing temperatures, the functional network of the *Coridius chinensis* gut microbiota transitions from an energy balance mode centered on lipid biosynthesis toward an energy metabolism framework driven by glycogen mobilization, glycolysis, and the pentose phosphate pathway. This transition occurs concurrently with the augmentation of nucleotide biosynthesis, the sulfur/methyl cycles, and cell wall biogenesis (Didion et al., 2021; Dittmer and Brucker, 2021). Such alterations not only optimize energy utilization efficiency but also provide the essential substrates for host antioxidant defense and epigenetic modulation, collectively sustaining physiological homeostasis during the diapausing state (Bernasocchi and Mostoslavsky, 2024). Metagenomic evidence underscores that, at diapausing temperatures, the *C. chinensis* gut microbiota achieves energy redistribution, membrane structural fortification, and antioxidant adaptation by modulating multiple pivotal pathways–including carbohydrate metabolism, nucleotide metabolism, sulfur/methyl cycling, and lipid synthesis–thereby elucidating the microbiological foundations of temperature-driven physiological mechanisms in diapause (Enriquez and Visser, 2023).

### 3.3 Fat body metabolomics reveal energy reserves and stress-resistant accumulation

**FIGURE 8:**
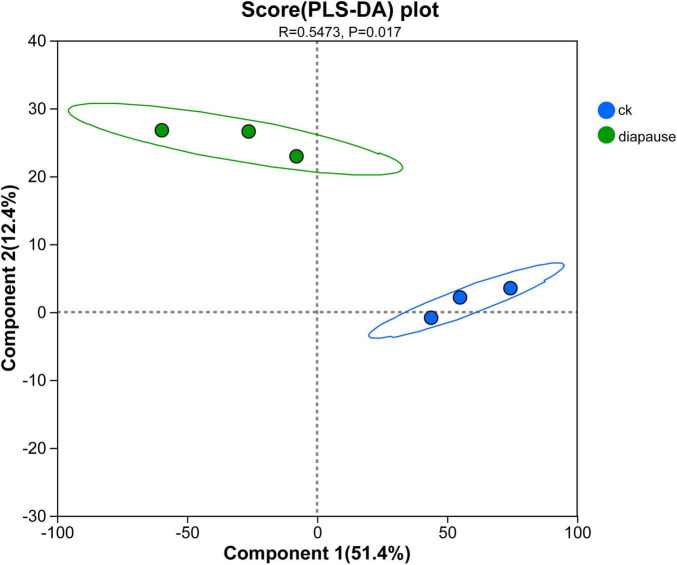
Principal Component Analysis (PCA) score plot.

**FIGURE 9:**
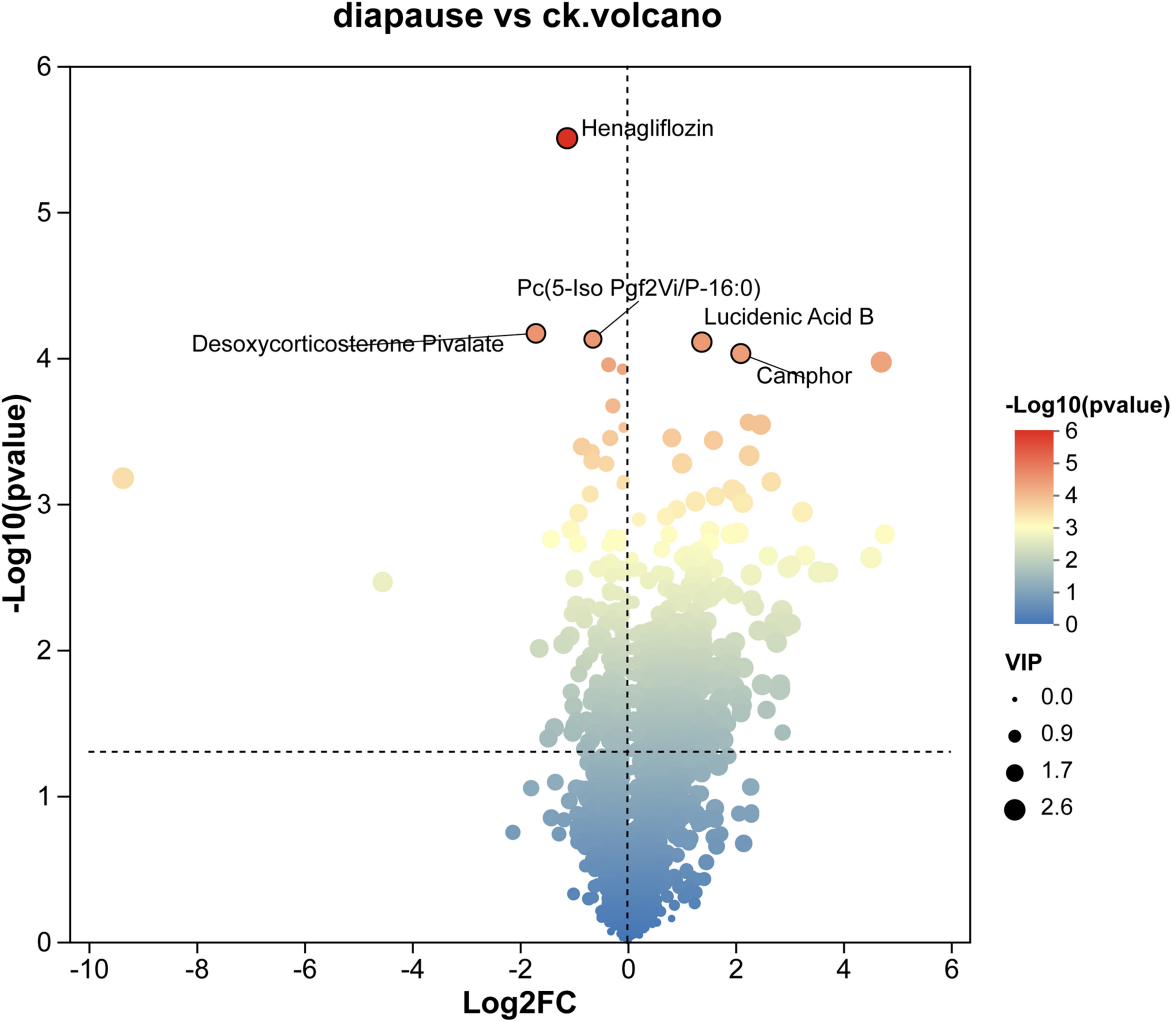
Volcano plot of differential metabolites.

**FIGURE 10:**
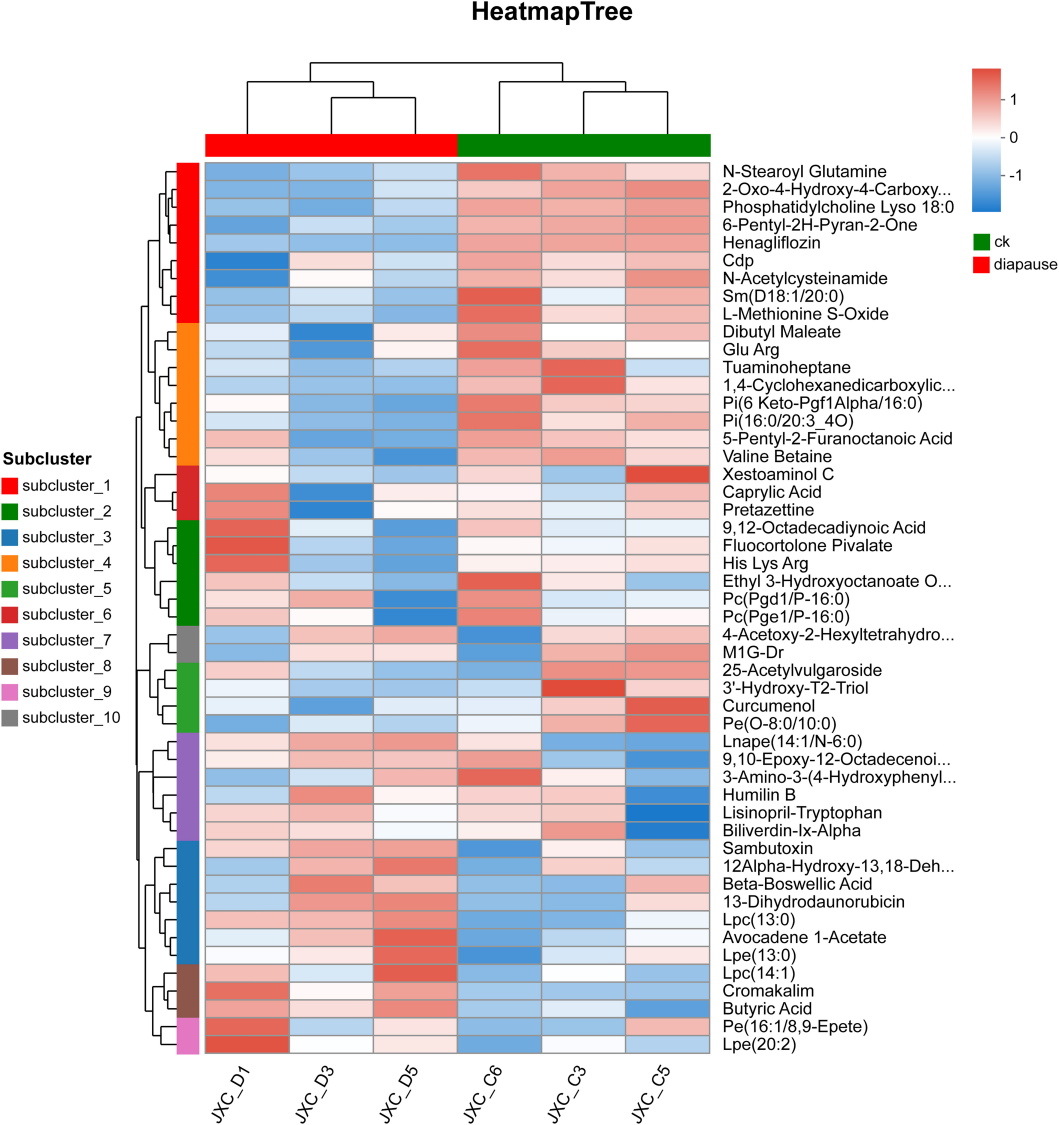
Hierarchical clustering heatmap.

**FIGURE 11:**
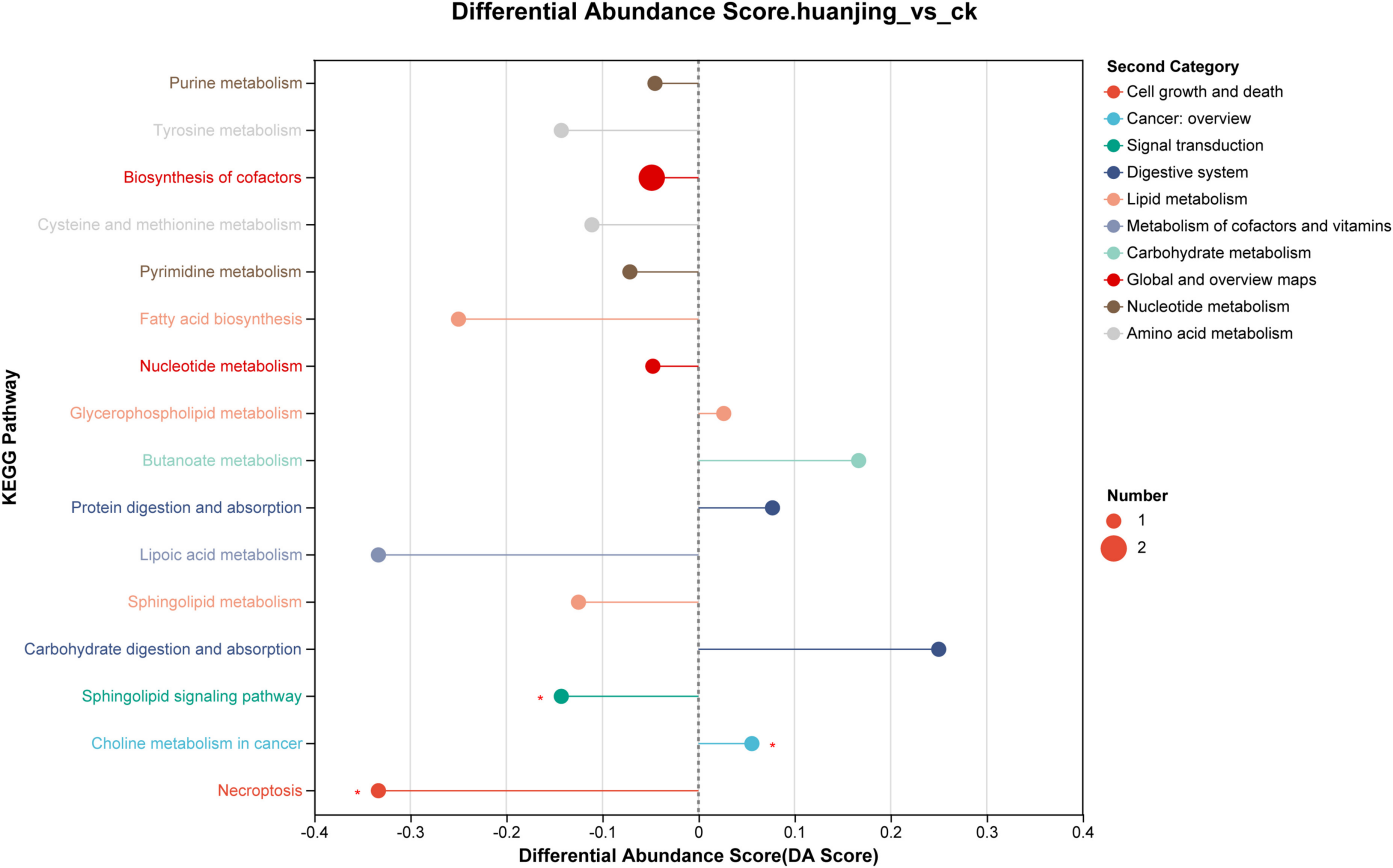
Kyoto Encyclopaedia of Genes and Genomes (KEGG) pathway enrichment analysis.

Principal Component Analysis (PCA) unveiled a pronounced remodeling of the metabolic landscape during the diapause process in *Coridius chinensis* (Figure 8). Specifically, samples from the diapause group deviated significantly from those of the ambient control group, whereas termination-phase samples clustered between the two; this indicates that low-temperature treatment induces a systematic drift in the fat body metabolic profile, which partially recovers upon re-warming, suggesting the reversibility of the diapause induction-termination cycle (Batz and Armbruster, 2018; Huang et al., 2022; Shao et al., 2024). Utilizing an OPLS-DA model (seven-fold cross-validation, R^2^ = 0.91, Q^2^ = 0.82) and applying the criteria of VIP > 1.0 and *p* < 0.05, a total of 189 significantly differential metabolites (SDMs) were identified, comprising 103 up-regulated and 86 down-regulated species (Batz and Armbruster, 2018; Huang et al., 2022). The volcano plot (Figure 9) demonstrated that the up-regulated metabolites predominantly included fatty acids (palmitic acid and oleic acid), polyols (e.g., trehalose and glycerol), and antioxidant-related substances (e.g., glutathione precursors and ascorbic acid derivatives); conversely, glycolytic intermediates (glucose-6-phosphate and lactate) and choline metabolites (choline and glycerophosphocholine) were significantly down-regulated. The overall metabolic trend manifested as a transition in the fat body from a “high glucose–high energy flux” state toward a “low glucose–high lipid–stress resistant” mode, whereby energy flux is redirected to sustain hypometabolic homeostasis. The metabolomic heatmap (Figure 10) illustrates a significant up-regulation of various fatty acids and phospholipid metabolites within the diapause group, characterized by the increased accumulation of long-chain fatty acids such as butyric acid, caprylic acid, and 9,12-octadecadiyonic acid. This indicates that upon entering diapause, *Aspongopus chinensis* redirects energy flux toward lipid sequestration by down-regulating carbohydrate metabolism. These fatty acids serve not only as the primary energy substrates during months of metabolic depression in diapause but also as precursors for the synthesis of signaling molecules (Hutfilz, 2022). Concurrently, *A. chinensis* preserves membrane integrity and functionality at extreme low temperatures by increasing the proportion of unsaturated fatty acids within membrane phospholipids to prevent phase transitions (from liquid-crystalline to gel phase), a process termed “homeoviscous adaptation” (Zhou et al., 2022). Kyoto Encyclopaedia of Genes and Genomes (KEGG) pathway enrichment analysis (Figure 11) revealed 13 significantly enriched pathways (*p* < 0.05, Impact > 0.1). Among these, the up-regulated pathways encompassed purine metabolism, fatty acid biosynthesis, glycerophospholipid metabolism, sphingolipid metabolism, butanoate metabolism, and the biosynthesis of cofactors; the down-regulated pathways were primarily associated with carbohydrate digestion and absorption, choline metabolism, and glycolysis. Notably, the significant enhancement of tyrosine metabolism and catecholamine biosynthesis pathways suggests that the fat body may participate in neurotransmitter-like signaling regulation during diapause, potentially establishing a feedback loop with cerebral JH/insulin signaling (Arrese and Soulages, 2010; Yin et al., 2025). Specifically, the augmentation of fatty acid and glycerophospholipid metabolism provides long-term energy reserves for diapause; the up-regulation of purine metabolism and glutathione-cofactor synthesis enhances antioxidant capacity and cellular repair mechanisms; and the activation of tyrosine–catecholamine metabolism implies that the fat body may engage in diapause regulation via metabolic-hormonal signaling. In summary, the fat body metabolome reveals the suppression of energy metabolism alongside the intensification of storage metabolism, the activation of antioxidant and membrane-lipid protective systems, and a putative coupling with neuro-endocrine networks during diapause. The systematic remodeling of these metabolic pathways provides the biochemical foundation for *C. chinensis* to maintain physiological homeostasis and ensure high survivability under low-temperature stress.

## 4 Conclusion

RNA-seq analysis demonstrated that under frigid conditions, brain tissues exhibit significant enrichment in neurotransmitter- and endocrine-related pathways (e.g., TRP sensation, cholinergic/5-HT synapses, and GnRH secretion); concomitantly, genes involved in the insulin/FOXO axis and lipid metabolism were up-regulated, whereas those pertaining to carbohydrate metabolism were down-regulated. These findings align with the classical paradigm of insect diapause, wherein the juvenile hormone (JH)/20-hydroxyecdysone (20E) and insulin-like peptide (ILP) axes synergistically suppress anabolism while promoting energy sequestration; the interplay between these hormonal pathways facilitates the inhibition of growth and reproduction, a reduction in metabolic rates, and an enhancement of stress tolerance, thereby underpinning both the induction and maintenance of the diapausing state. This regulatory framework has been extensively validated across multiple species and reviews, with insulin signaling identified as a pivotal node in the bioenergetic regulation of diapause (Hahn and Denlinger, 2011). Integrated with the current fat body metabolomic data–characterized by the up-regulation of fatty acids/phospholipids and cryoprotective/antioxidant metabolites alongside a decline in glycolytic intermediates–it is suggested that thermal cues are transduced through the brain-endocrine network to redirect energy flux from carbohydrates toward lipid reserves and activate stress-related metabolism, ultimately manifesting as the “hypometabolism–high sequestration–stress resistance” diapause phenotype. This bioenergetic explanation of “developmental arrest and energy conservation” is in high accordance with established literature (Hahn and Denlinger, 2011).

Metagenomic profiling reveals that at diapausing temperatures, the functional landscape of the gut microbiota transitions from lipid biosynthesis toward a framework dominated by glycogen mobilization, glycolysis, and the pentose phosphate pathway (PPP), accompanied by the concomitant intensification of nucleotide metabolism, sulfur assimilation, and the methyl cycle. Prior investigations have established that the architecture and functionality of the insect intestinal community undergo profound remodeling during diapause or dormancy; specifically, the microbiota can replenish energy substrates and reducing power for the host via the provision of metabolites such as short-chain fatty acids (SCFAs), or modulate inflammatory-immune homeostasis through receptor-mediated or epigenetic pathways. Within models such as the cabbage beetle, the microbial composition exhibits significant divergence between the pre-diapause and non-diapause phases, suggesting that the microbiota contributes to metabolic pre-setting prior to the onset of diapause (Liu et al., 2016). From a mechanistic perspective, SCFAs have been demonstrated to regulate immune and metabolic pathways through G-protein coupled receptors (GPCRs) and histone deacetylase (HDAC) inhibition; although these findings are predominantly derived from vertebrates and model organisms, they provide a generalized mechanistic framework for such interactions. These findings resonate with our observations of enhanced sulfur/methyl cycling (reflecting antioxidant and epigenetic regulatory potential) and intensified PPP (ensuring NADPH supply), collectively providing a metabolic foundation for the host to sustain cellular repair and redox homeostasis under low-temperature stress (Zhang et al., 2024).

Fat body metabolomic profiling revealed the significant accumulation of trehalose, glycerol, fatty acids, and glutathione-related metabolites, concurrently with a decline in glycolytic intermediates and choline metabolites; this illustrates that energy flux is suppressed and prioritized toward nutrient sequestration and stress tolerance during diapause. Established reviews have underscored that cryoprotective solutes, such as trehalose and glycerol, markedly enhance cold survivability by stabilizing membrane structures and inhibiting ice crystal formation, complemented by the recruitment of heat shock proteins (HSPs) and antioxidant systems. The molecular and metabolic “fingerprints” identified in this study are consistent with these established paradigms, further suggesting that antioxidant defense, membrane-lipid homeostasis, and hypometabolism represent parallel, rather than mutually exclusive, physiological strategies. Accordingly, we postulate the following working model: cold signals stimulate the cerebral thermosensory and circadian networks alongside the neuropeptide-hormonal axis (JH/20E/ILP), leading to the down-regulation of carbohydrate metabolism and the up-regulation of lipid and stress-related pathways. This is accompanied by the functional remodeling of the gut microbiota (involving carbohydrate, nucleotide, and sulfur/methyl metabolism), which provides systemic metabolic and immune support; ultimately, multi-organ coordination is achieved through metabolite and hormonal feed-forward/feedback loops to regulate the induction, maintenance, and termination of diapause. This proposed model aligns with the hierarchical integration framework of “chronobiological-endocrine-metabolic” regulation (Helfrich-Förster, 2024).

This study has established a systemic molecular–metabolic network model for diapause in *Coridius chinensis*, providing a robust empirical foundation for understanding the multi-organ synergistic mechanisms underlying insect diapause. The findings offer theoretical support for the predictive modeling of overwintering and the development of ecological pest management strategies; furthermore, they identify potential avenues for RNAi target screening, micro-ecological interventions, and the artificial manipulation of diapausing states. Future research should incorporate emerging technologies–including single-cell transcriptomics (scRNA-seq), spatial metabolomics, and metabolic flux analysis–to further elucidate the spatiotemporal dynamics and cellular heterogeneity associated with diapause.

## Data Availability

The original contributions presented in the study are publicly available. Metabolic group raw data can be found at https://ngdc.cncb.ac.cn/bioproject/browse/PRJCA061696 and original data of transcriptome and metagenome https://www.biosino.org/node/project/detail/OEP00006964.

## Footnotes

1
https://www.biosino.org/node/project/detail/OEP00006964
